# Caring touch in intensive care nursing: a qualitative study

**DOI:** 10.1080/17482631.2022.2092964

**Published:** 2022-06-27

**Authors:** Lise Sandnes, Lisbeth Uhrenfeldt

**Affiliations:** Faculty of Nursing and Health Sciences, Nord University, Bodø, Norway

**Keywords:** Critical care nursing, Nurse-Patient relations, culturally competent care, dignity, ethics, touch, clinical wisdom

## Abstract

**Background:**

Historically, caring touch was integrated in targeted nursing acts as shoulder massage, calming patients or to check vital parameters by touching the patient`s skin. However, this phenomenon in intensive care nursing still lacks convincing descriptions. Caring touch is an important part of being an intensive care nurse and confirming the patient`s dignity. To touch the patient`s skin is a common nursing act, but not much spoken of. Caring touch on the patient`s chin, holding hands or giving a hug has earlier been called e.g., non-procedural touch.

**Purpose:**

Explore the meaning of caring touch as it appeared for Norwegian intensive care nurses.

**Methods:**

Secondary analysis of data from qualitative, individual semi-structured interviews. Eight experienced intensive care nurses at public Norwegian non-university hospitals. Registered by the Norwegian Center for Research data NSD December 2014. ID 41164.

**Findings:**

Data analysis revealed one main-theme: The speaking body, with four sub-themes 1) Eyes and facial expressions, 2) Patients emotional expressions, 3) Closeness and distance, 4) ICU nurses’ emotional responses.

**Conclusion:**

Caring touch is a silent way of showing culturally competent care and establish or continue nurse-patient relationships in intensive care units. Caring touch contributes to heighten ethical dimensions of dignity in intensive care nursing.

## Introduction

Intensive care patients experience dramatic life changes when they develop a sudden critical illness. They may experience fear, loneliness and isolation in an unfriendly, technical hospital environment (Fredriksen & Ringsberg, 2007). Intensive care patients are admitted for 3–4 days on an average (Moitra et al., 2016). Amid high procedural demands, the individual intensive care unit (ICU) nurse sets the bedside standards. However, the shortage of time may challenge ICU nurses’ prioritization (Tønnessen et al., 2020). Organizational conditions influence nurses’ capacity to engage in building relationships with patients and creates ethical challenges about communicating nursing errors (Bridges et al., 2013; Ghezeljeh et al., 2021; Usberg et al., 2021).

The public tax-funded healthcare system in Norway expects nursing to encompass dignified, moral, ethical and humane care (Martinsen, 1996). ICU nurses require insights and skills to discover nearly invisible signs among the patients, act and/or prevent actions in each situation (Benner, Hooper-Kyriakidis et al., 2011b). ICU nurses’ decision-making process is highly complex and involves touching the patient’s skin (Aitken et al., 2008). Caring touches such as stroking the patient’s chin or holding his hand are important to preserve the patient’s dignity and communicate safety and care (Bundgaard and Nielsen, 2011; Playfare, 2010; Hov et al., 2007).

Dealing with critically ill patients may induce both positive and negative emotions among ICU nurses, which may become challenging over time (Magro-Morillo et al., 2020). The multi-layer protective clothing during the COVID-19 pandemic prevents caring skin-to-skin touch (Nist et al., 2020). However, the ICU patient’s need for a caring touch is essential for the nurse-patient relationship.

ICU nurses touch patients’ skin to soothe and comfort them, and this caring touch has been described as non-procedural, non-task oriented, expressive, protective, tactile and affective massage (Almerud et al., 2008; Gleeson & Timmins, 2005). Patients may experience the nurse’s caring touch as positive and comforting. However, a general touch may for the patient be associated with pain, bad experiences, and negative presence (Borch & Hillervik, 2005). Previous literature on ICU patients and nurses has mainly focused on the clinical effectiveness of touch as e.g., massage in order to relieve pain (Gleeson & Timmins, 2005). However, here caring touch is characteristic of a person-oriented care, in which building the nurse-patient relationship, establishing mutual means and ends and promoting well-being are central to the nurse’s attention (Uhrenfeldt et al., 2018). Touch in this article is an expression of a fellow human openness to another person`s vulnerability, dignity and overall situation. Caring touch means that one fellow human being is employed for his job, but at the same time reaches out to another human being on the basis of his own understanding of fellow human dignity, care, duty and conscience.

A human being is admitted to a high-tech hospital and receives the necessary treatments. The loving care that comes without a purpose, but from an open compassionate desire to offer individual dignity, can awaken hope in the midst of a sense of hopelessness. However, it requires the surplus to be aware of this necessity, and to venture forward and to make oneself available in the situation.

Some theoretical milestones that have influenced nurses’ understanding of caring touch since the 1960s is presented to identify the historical roots and the present knowledge gap in ICU nurses’ care.

### Caring touch in the 1960s and 70s: historical reflections

Virginia Henderson’s (1897–1996) guidelines were adopted globally by the International Council of Nurses (Henderson, 1997). Her guidelines referred to Maslow’s need hierarchy and emphasized on helping the patient achieve good health, independence or a peaceful death (Maslow, 1943). Basic human needs guide nurses to support patients in maintaining their physical, psychological and emotional balance (see Box 1).

**Table:** 

BOX 1: Henderson suggested 14 basic needs that guide nursing practice: 1) Breath. 2) Eat and drink. 3) Eliminate body wastes. 4) Move. 5) Rest and sleep. 6) Get dressed. 7) Keep a normal body temperature. 8) Keep the body clean. 9) Avoid dangers in the surroundings. 10) Communicate and express emotions, needs and fears. 11) Practice religion. 12) Have tasks that give a feeling/sense of accomplishment. 13) Play and participate in recreation. 14) Learn and discover.

To maintain a patient’s physical, psychological and emotional balance, Henderson adds that:

‘The unique function of the nurse is to assist the individual, sick or well, in performance of those activities contributing to health or its recovery (or peaceful death) that he/she would perform unaided if he/she had the necessary strength, will or knowledge. And … gain independence as rapidly as possible’ (Henderson, 1997).

Henderson guided nurses to comfort the patient through their caring presence and touch, justified as help for a patient undergoing pain and discomfort (Henderson, 1991).

Dorothea Orem (1914–2007), who proposed the *Self-Care Deficit Nursing Theory*, defined nursing as “a human service concerned with the health and well-being of individuals and groups.” (Orem, 2001)The priority was to help patients become capable of fulfiling their self-care demands through five methods in which one person compensates or overcomes the limitations of others to act for themselves in their daily life. Orem separated the methods into five groups (see Box 2).

**Table:** 

BOX 2 Orem’s five methods include: 1) Acting or doing for another. 2) Guiding and directing. 3) Providing physical or psychological support. 4) Providing and maintaining an environment that supports personal development. 5) Teaching.

The nurse acts as the patients would have done if they could. The goal is for the patient to take over and regain independence since “acting for another may be gradually replaced by methods of guiding, supporting and teaching another” (Orem, 2001). Orem highlights the importance of offering care in a respectful and trusting way which may lead to caring touch. The relationship holds an agreement to receive and provide nursing. The nurse has a social responsibility towards the comatose patient along with his needs for contact, observation and care, which is influenced by his situation (Orem, 2001).

Joyce Travelbee (1926–1973) described nursing as an interpersonal process to improve the patient’s health by assisting the individual, family or community (Travelbee, 1971). Travelbee warns against reducing the ill human beings to the term patient and treating them as a task. The patient’s needs are at the forefront of nursing, since there is no substitute for talking with the ill person. On the other hand, the nurse should be able to grasp the patient’s need for assistance without the patient’s request (Travelbee, 1971)

“Each contact the nurse has with the recipient of her care can be a step leading in the direction of a human-to-human relationship. This is especially true if, in each contact, the nurse intentionally strives to know the recipient of her care and to ascertain and meet their needs.” (Travelbee, 1971)

Although Travelbee’s clinical interest was in psychiatric patients, her theory has been used broadly in nursing. No specific focus on caring touch was identified in her text; however, other scholars have interpreted the relationship model into palliative care and nurses’ intervention in the patient’s suffering process (Parola et al., 2020)

In summary, nurse scholars in the 60s and 70s mostly addressed the basic human needs. Maslow influenced both Henderson’s and Orem’s nursing theories. Travelbee elucidated the scholarly interest in building a caring nurse-patient relationship.

### Caring touch in the 80s and 90s

atricia Benner (1942–) explored nurses’ understanding of what the patient communicates with his/her body, which is possible when the nurse feels involved in the patient’s situation (Benner et al., 2011a; Benner et al., 2009). ICU nurses develop expertise with a clinical grasp and forethoughts of the patient’s situation. Benner emphasizes the importance of the nurse protecting the patient`s dignity in times of vulnerability. To achieve this, the nurse uses language, listens and notices the air and the person’s scent in addition to caring touch (Benner et al., 2011a). Nurses’ cumulative wisdom involves a clinical judgment and scope to prevent patients’ acute risks and future problems (Benner et al., 2011a; Benner & Wrubel, 2001). Caring touch is described in the breaks that occur between procedures.

Kari Martinsen (1943–) and Katie Eriksson (1943–2019) developed their theories of caring in different Scandinavian countries and based on different ideas (Arman et al., 2015). Martinsen described care as a moral practice that is part of the human community (Martinsen, 2012). She raised questions on whether the nurse’s attention is drawn to technical procedures and treatment rather than caring acts and fundamental human needs. She urged nurses to “See with the eyes of the heart” and be sensitive towards the patient’s vulnerability in the situation (Martinsen, 1993). Her values stem from Protestant ethics and Løgstrup’s phenomenological view of life where all human beings are part of each other’s world and depend on each other.

Eriksson presented a caritative theory in nursing that highlights love, charity, faith, hope, tending and playing. Tending care is characterized by warmth, closeness and touch. Maintaining the patient’s dignity is an ethical responsibility of ICU nurses and other caregivers (Eriksson, 1987)(Martinsen, 1993). Dignity is a basic concept in caritative caring ethics.

‘Human dignity is partly absolute dignity, partly relative dignity. Absolute dignity is granted the human being through creation, while relative dignity is influenced and formed through culture and external contexts.’ (Lindström et al., 2021)

However, Eriksson also emphasized the nurses’ possible role in the patient’s suffering:

“Suffering violates human dignity, and to violate human dignity is to cause suffering. Suffering is in fact not worthy of human beings. Whenever a person suffers, he feelsviolated” (Eriksson, 1995)

Caring for the body has no specific emphasis in Eriksson’s theory; however, mental care has been inspired by Catholic metaphysics (Uhrenfeldt, 2021)

In summary, the 80s and 90s addressed scholarly engagement in the patient as a person and a fellow human being granted dignity and expressing fears, hopes and wishes.

### Theoretical frame: caring touch in the 2000s

The European Academy of Caring Science (EACS) in 2004 (European Academy of Caring Science (EACS), 2021) advanced caring science from both a European and interprofessional perspective (Albarran et al., 2011). Initially, this academy led scholarly members from Sweden (Dahlberg & Segesten, 2010) and the UK (Galvin, 2010), and later from Denmark, Greece, and Norway. The carer assists the patient’s movement from suffering to well-being and seeks to involve the patient’s perspective (Dahlberg & Segesten, 2010). Galvin described existential well-being as a balanced dwelling-mobility keeping the patient’s dignity as an important part of the caring effort (Galvin, 2010). In Scandinavia, caring science combines a philosophical humanistic approach that stands out from an earlier more religious orientation seeking to humanize the technical, procedural and instrumental knowledge often seen in ICUs (Arman et al., 2015; Galvin, 2010). Caring touch is a unique caring act that promotes each patient’s health and well-being (Airosa et al., 2016). Touching the skin in different ways creates meaning as it stimulates the patient’s (apostrophe after S in ”patients”) openness for care and presence in a meaningful context while being validated as human beings. The phenomenon of caring touch is not generally highlighted through the historical periods in which nursing theory has dealt with care. It may be because it was a matter of course and implied, but it may also be because it has not been an area where there was a demand for development, and it was therefore not linguistic. In our study, touch is a universal human activity that occurs when people are in any kind of relationship. Caring touch is a spontaneous, unplanned, kind action that occurs without a conscious purpose in a professional caregiver. We therefore exclude the type of touch performed with a professional or private purpose or which is part of a strategic action (World Medical Association, 2013).

With this study, we further want to linguize the experience of nurses with caring touch. Therefore, we explore the meaning caring touch has for Norwegian intensive care nurses with the main question: what motivates the ICU nurse to touch the patient with a caring touch when there is no procedure to be done.

## Materials and methods

### Study Design

This empirical study employed French philosopher Paul Ricoeur’s understanding of spoken language between, for example, an interviewer and informant. Ricoeur’s hermeneutic philosophy of interpretation (Dreyer & Pedersen, 2009; Ricoeur, 1999) was translated into a three-step methodological guide starting with a naïve reading of the transcribed interviews, followed by a structured condensation of meaning units and a comprehensive discussion (Ricoeur, 1999). We initiated a secondary stepwise interpretation of unpublished data from mid-twenties (Lindseth & Norberg, 2004) to further the dialogue with ICU nurses during specialist training.

## Participants

The study included eight ICU nurses with 5–30 years of experience (seven women, one man; see, Table I). The participants were employed at two ICUs in rural non-university Norwegian hospitals. First author contacted charge nurses at ICU departments who handed out an information letter to the specialist nurses with experience of five years or more. To participate, each nurse signed a document stating that they were informed, that their response was anonymous and that they could withdraw their commitment to participate at any time or for us to use their information but none of them expressed such a wish.

**Table I.** Participants included in the interview (n = 8).

ID	Non–university hospital 1–2	Gender (F/M)	Years of nursing experience (11–34)
A	1	Female	34
B	1	Female	11
C	1	Female	22
D	1	Female	30
E	1	Female	17
F	2	Male	27
G	2	Female	15
H	2	Female	16

## Data collection

The first author conducted 30–50 minutes semi-structured interviews, including open-ended questions, with all participants (Kvale & Brinkmann, 2009). The main question was “What initiated or triggered touch like holding the patient`s hand or stroking his cheek, when there was no procedure to be done?” Additional questions were e.g.,: What thoughts do you have about touch? Do you touch every patient in the same way? Most interviews were conducted in an undisturbed and quiet room in the ICU; however, two interviews were conducted at the nurses’ homes at their request. The interviews were audio recorded and transcribed verbatim into texts. The first author transcribed all interviews, they were anonymized during this process as the participants` names were removed and replaced with an alphabetic letter.

## Data analysis

The interviews were analysed using Lindseth and Norberg`s three-phased phenomenological-hermeneutical method (Lindseth & Norberg, 2004). In the naïve reading, all texts were read repeatedly by both authors to reach a mutual first overall impression of the content. This first, open and naïve understanding was written by the first author and then discussed with the second author so that both authors agreed and contributed based in the text. In the structural analysis (see, Table II), the text was read repeatedly by the first author until a structure of quotes, condensed meaning units showed patterns and similarities. Categories were created and patterns organized in themes. The analysis was done manually using Word © documents on tablets for each interview text, creating one new document for each subtheme. Through close collaboration, both authors identified one main overarching final theme with four subthemes (Lindseth & Norberg, 2004). The last step in the analysis was re-reading the original transcripts to reflect on our findings in relation to the research question (Airosa et al., 2016). This was a secondary analysis (Heaton, 2008) where a new research question was being asked to existing data. Both authors had a role in interpreting the data in the light of the concept of dignity, and following the three-step analysis process of (Lindseth & Norberg, 2004)

**Table II.** Illustration of the structural analysis.

Quotes from text	Condensed meaning units	Sub-themes	Main theme
You see it in their eyes, it’s hard to explain. It’s a kind of wandering look that follows you around the room. My gut feeling tells me not to touch. (B)	You see it in their eyes, it’s hard to explain, it’s a kind of wandering look. My gut feeling tells me.	Eyes and facial expressions	The speaking body
To touch the patient can do good even though he doesn’t seem open to it and says he doesn’t have pains. You can see that the body screams out “I’m not good”. You can see the shoulders are raising. If you then put your hand on his shoulder and tell that you can see he has pains, it can be a kind of relief and he can say that “Yes, I do have pains”. (E)	Touching the patient can be good even though he says that he does not have pain. The patient’s body screams out “I’m not good” and his shoulders are raising.	Patients’ emotional expressions	The speaking body
If the response from the patient is to pull his hand back or show in other ways that he doesn’t want any contact, I try to be careful. Maybe I just touch the bed … (D)	If the patient pulls his hand back, I’m careful not to scare him. Maybe just touch the bed.	Closeness and distance	The speaking body
If you reach a goal, if you feel that the patient could manage because you are there and what you do, because you show empathy and care and touch their skin. If you feel that what you do is useful, that is a good feeling. (A)	If you feel that the patient could manage because you show empathy, care and touch, that is a good feeling.	ICU nurses’ emotional responses	The speaking body

The first author, an experienced ICU nurse with a master’s degree in intensive care nursing and the second author, a trained nurse and expert qualitative researcher, had a critical dialog throughout the analysis.

### Ethical considerations

The study was registered at the Norwegian Center for Research Data (NSD) in December 2014 (ID 41164; World Medical Association, 2013). Before the interviews, participants were informed about the study’s purpose, anonymity of their contribution, and their right to withdraw their written consent anytime. Each participant signed an informed consent form before the interview started. No participant withdrew from the study.

## Results

The findings contain a naïve understanding of the text and a structural analysis with one main theme (the speaking body) and four sub-themes (eyes and facial expressions, patients’ emotional expressions, closeness and distance and ICU nurse’s emotional responses).

### Naïve understanding

The naïve reading revealed that ICU nurses seldom discussed caring touch. The ICU nurses lacked words to describe caring touch; however, they expressed how caring touch was connected to their preservation of patients’ dignity. Caring touch is a part of building a relationship with a patient. ICU nurses argued that they sought to transfer comfort, hope and strength to the patients through their touch and support their dignity in a technical ICU environment. The touch could replace words or reinforce spoken words.

ICU nurses described how they read the situation in the room. They sensed the atmosphere and assessed the patients’ eye glances and body movements. ICU nurses assessed both the appearance of the patient’s skin and how it felt upon touching as an overall evaluation of the patient’s condition. Some patients produced a specific odour of fear and nervousness, which the ICU nurses interpreted as a need for a caring touch. Each time they touched a patient, they decided how and where to touch, since the touch could lead to either good or bad experiences for the patient. A caring touch may have a calming effect but can sometimes cause an aggressive patient’s deterioration. Although there were warnings to maintain a distance, ICU nurses sometimes interpreted that these patients needed a caring touch to open up and communicate their fears and troubles.

### Structural analysis

The structural analysis revealed one main theme and four sub-themes (Table II).

#### The speaking body

The speaking body is an expression from the ICU patients’ bodies that is sensed by the ICU nurses. In the relationship with the patient, a special “gut feeling” arises in the ICU nurse as a reaction to what the patient expresses. This non-linguistic message seems to prepare and guide the way the ICU nurse provides a caring touch to impart dignity and care. The expressions are presented under the following four sub-themes:

1) *Eyes and facial expressions*

The look in the patient’s eyes and their facial expression motivated the ICU nurse for a caring touch, as expressed by one nurse:

B: I look at how their faces and eyes appear. If they’re tense, look worried, if they’re sweating. They cannot always say something.

A worried or tense expression and a damp appearance were signals to offer a caring touch. It was easier to judge whether to touch if the patient was conscious, as one ICU nurse stated:

D: If they’re conscious, I can sense so much more. They give me signals with their eyes or speak.

If a patient asked to be touched, the ICU nurse could rely on their response through facial or bodily expressions or eyes. However, it is different with a comatose patient. The nurse aims to comfort the patient through a caring touch but needs to be aware of the boundary of the patient’s dignity and acceptance of caring touch. This judgment is possible if the patient is awake. Additionally, the patient’s facial expressions may reveal the need to avoid touching. Two ICU nurses stated:

F: Some (patients) give you a dark look. That’s unpleasant. They do not need to say that much, but you understand that you must not touch, you pull back a bit.

C: Sometimes, restless or aggressive patients become even more stressed upon being touched. Some also get this dark look in their eyes and become upset.

Therefore, looking in the patients’ eyes and their facial expressions could signal the ICU nurses whether to touch the patient. The way to ascertain this is difficult to describe, as stated by an ICU nurse:

B: To touch or not. You can see it in their eyes, which is difficult to explain. A kind of wandering look tells me if I must avoid touching. My gut feelings tell me.

The ICU nurse used her senses and experience along with the patient’s expressions. This combination leads to what the nurse calls a “gut feeling”; a kind of physical response in the ICU nurse’s body based on what she/he observes and registers. When the ICU nurse holds the patient’s hand, it can have a calming effect, as one nurse explained:

A: There was desperate expression on her face and sighing. She was so anxious about wearing the oxygen mask, but if I sat close to her and held her hand, she managed it.

The ICU nurse provided a caring touch in response to the patient’s desperate facial expressions and sighing. When the ICU nurse was close and attended by holding the patient’s hand, she could manage the unpleasant treatment.

2) *Patients’ vulnerability, anxiousness and emotional expressions*

Expressions of a patient’s feelings and signs of vulnerability seem to trigger a caring touch.

C: Vulnerability and touch are similar. These patients are vulnerable and alone. We cause discomfort, and they are anxious about what happens. Having a hand to hold does well in these situations.

Professional experiences explain that holding hands is what a patient needs for comfort, since a caring touch shows an empathic and dignifying side of the nurse’s care.

G: When I know a patient, I know more about what he or she needs and accepts. It varies from person to person, but you can soon read a patient’s needs.

To adjust the caring touch to each patient, the ICU nurse interpreted the patient’s emotional expressions and signals. This becomes easier when the nurse has spent some time with the patient. The ICU nurses’ sensitivity preserves the patient’s dignity when there is a choice. Sometimes, the signals can be compound.

E: Some patients have a tough exterior, but a caring touch can break down their defense mechanisms. They say they have no pain, but I observe pain from their eyes; they lie quite stiff in the bed and exude a special nervous sweaty odour. Words together with caring touch can open things up, and I understand what the problem really is.

A: If you think that the reason for him to show anger is that he is scared, then it is natural to touch him.

The ICU nurse’s caring touch was initiated by various senses, as she noticed fear and a special body odour from the patient’s body language. Anger is often interpreted as a signal not to touch; however, a caring touch was given when it was interpreted as a concealed signal of fear.

B: Patients rarely say: I am afraid! It’s something I perceive and see, then I think to myself; What would I have wanted if I was in the same situation?

When the patient showed his feelings, the ICU nurse considered herself to be in the same situation, which affected her caring touch and prevented loss of dignity.

E: His hand over his eyes, he also showed a sense of shame. He sent out many signals, and then it was all that crying and despair. When I see major emotional outbursts, it makes me want to give a caring touch.

After observing many patient’s emotional outbursts, the ICU nurse’s response was to provide a caring touch.

3) *Closeness and distance*

Closeness and distance are important elements in caring touch.

G: When you sit down, spend some time together, then you understand what the person is trying to express. The first touch may be on the hand, you try some touching, and then you assess what is accepted. Touching will bring you closer. And if you`re close, it is more natural to touch.

Caring touch seems to establish an impression of closeness between ICU nurses and anxious patients. The ICU nurse creates a balance between proximity, distance and caring touch in relation to the individual situation based on a continuous interpretation of signals from patients. This requires spending time. During an acute illness, a close dignified relationship can grow.

H: I sat with him for an hour after the doctor’s message. I held his hands so that he could feel trust and confidence. He then managed to talk about his fear. Caring touch was a natural part.

The patient was given time and opportunity to share feelings and worries without loss of dignity. The special closeness gave the ICU nurse a deeper understanding of the patient, and it became natural for her to provide care. In contrast, caring touch is sometimes deliberately avoided, as stated by one ICU nurse:

B: These shifts when you are always in a run. I can see that the patient needs to talk, and if I sat with him and held his hand, it could give him room to talk about his worries. I avoid caring touch when the telephone is constantly ringing, and all I can think about is all I have to do before my shift ends. However, it does not feel good, it really does not.

The ICU nurse was ashamed to talk about her avoidance of caring touch. Sometimes, keeping a distance seems necessary to manage all the tasks scheduled within working hours. Caring touch requires time which is not always available for ICU nurses. However, not all patients appreciate the closeness provided by touch, as explained by an ICU nurse:

F: If the patient is aggressive and difficult to relate to, I keep my distance. I always try to get close to the patient, but they are not equally responsive.

ICU nurses maintain distance when patients are angry and signal to stay away. However, the nurses still attempt to get closer to the patient, which indicates that caring touch is seen as important despite the patient’s unapproachable expressions.

4) *ICU nurses’ emotional responses*

The patient may trigger ICU nurses’ emotional responses. One ICU nurse explained how emotions are triggered:

H: Before I touch, I sense the atmosphere around the patient, the feelings the patient has, and the unease he feels. I get emotionally touched without any words being said.

The ICU nurse sensed the atmosphere in the room based on the patient’s situation and feelings, which seemed to move the nurse emotionally to support the patient’s dignity by providing a caring touch. An ICU nurse elaborated:

E: Why you touch and why you get emotionally touched, it is empathy. To put yourself in another person’s situation … You try to understand what is going on, it’s a part of being a nurse. The other person’s vulnerability and the need for help makes me emotionally touched.

The patient’s situation impacts the ICU nurse, who gets emotionally touched. This evokes a desire to help and support the patient by providing a caring touch. To do what is best for the patient in each situation, empathy and a sincere desire to maintain the patient’s dignity throughout the illness and suffering is required. One ICU nurse had tears in her eyes when talking about her emotional responses:

B: You do get affected. You share joys and sorrows, with a stranger, that connects you closely and I`ve shed some tears over the years. I do not feel ashamed of giving a patient a hug; they thank me for being so physical.

The ICU nurse allowed herself to be moved by the patient’s situation and feelings, which initiated a caring response. Some patient situations may trigger memories of the ICU nurse’s own life.

H: If there are situations like my own private life, I get more emotional. I felt the fears the patients felt, and I tried to give the patients what made me feel more at ease. Such settings affect me as much every time and make it difficult to move on to the next patient.

Situations similar to the ICU nurse’s private life seemed to reinforce the emotional response. This motivated the ICU nurse to touch the patient in the same way as if comforting herself. The fight for a dignified response is both challenging and emotionally exhausting, as the ICU nurse needs a break before attending the next patient. One ICU nurse talked about a deep wish for the patients to make it:

A: I mostly touch patients that signal a need. Despair, helplessness, that they are afraid. I want them to make it. I need to use the whole of me, everything I can in order to help them.

The ICU nurse constantly sought the patient’s signals to adjust the caring touch accordingly. The phrase “the whole of me” implies the need for different kinds of knowledge. For example, ethics involve professional nursing expertise, building relationships and experience as a fellow human being. These types of knowledge together dignify the ICU nurses’ way of providing a caring touch.

## Discussion

Our main question is: what motivates the ICU nurse to touch the patient with a caring touch when there is no procedure to be done. A search for historical evidence on caring touch did not provide an answer from a specific nursing theory, possibly because nursing till the 1980s took the nurses’ non-procedural touch to comfort patients for granted. In the 1980s, technical procedures in ICU nursing increased rapidly along with the time pressure on nurses. Orem’s three levels of nursing assistance for patients’ needs introduced the act of assisting others in managing self-care based on their individual needs and become capable of enhancing their well-being in their present situation. From the 1990s until today the importance of nurses protecting the patient`s integrity and dignity is emphasized by several nurse theorists (Benner et al., 2009; Eriksson, 1987; Galvin, 2010; Martinsen, 2012). Our participants search for words to describe what is going on as “it`s something I receive and see” (participant B) or an embodied feeling if caring touch is a right action now, if it “feels natural to touch” (participant A, G, H).

Well-being as an existential contribution within caring science and theory becomes clear in connection with Galvin and Todres interdisciplinary research (Galvin, 2010; Galvin & Todres, 2015). It is understood from the person’s experience of existentials such as mood, relationships, identity, body, temporality, or spatiality, which influences the individual’s perception of their dignity and situation (Galvin & Todres, 2015). Being a patient in modern technical ICU departments narrows the relationship, mood, and identity. However, the bedside ICU nurse enhances dignity within a temporal period,(Galvin, 2010) sometimes by providing a caring touch as illustrated by our study.

According to Galvin seven kinds of dignity are interrelated (Galvin & Todres, 2015). The human being is already valuable in oneself; however, dignity constitutes the deep value of each human`s being in the world. When dignity is divided into seven areas, *Spatial dignity* emerges. It includes one`s space in the world, a feeling of being at home. Then there is *Temporal dignity* it is associated with one`s sense of history, possibility, to the present temporal rhythm of night and day, seasons and the phases of one`s life. As the next comes *Embodied dignity*: to relate to an embodied heritage and continuity being-in-the-world. A sense of the bodily “I can” and “I am” and a painful awareness of a bodily privacy if invaded or ashamed. *Interpersonal dignity* is the deepest appreciation of our common humanity and a gift one another share when “mattering” to one another. This can also be misused and harmful to others. *Identity dignity* is related to how things are for us, spatially, bodily, temporally, in mood. This self-gathering is core to our sense of identity and has deep implications for dignity. When challenged, it is a threat to our identity. *Mood dignity*: can be carried in many moods; in solemn sadness, celebratory joy, poignant love, defiant determination and simple “here-I-am peacefulness”. *Finitude dignity*: A dignity experience attuned to the potential of our existence to “not be”; The potential of personal death. The dignity of embracing life`s limits.

### Our findings discussed in relation to seven kinds of dignity

The temporal dignity is in place when non-procedural caring touch is ICU nurses’ emotional reaction to a patient’s anxiousness, vulnerability and mood and it also connects to serve the identity dignity. Offering relationships and acknowledging the patient’s spatial dignity with humanized care reach out to overcome the risk of dehumanizing (Galvin & Todres, 2015) in a highly technical environment (Almerud et al., 2008)

Exploring the meaning of caring touch in a Norwegian public-funded healthcare system implicates that caring is also interpersonal in appraising the person’s dignity (Uhrenfeldt et al., 2018). Our main theme, i.e., the speaking body, and four sub-themes, including eyes and facial expressions, patients’ emotional expressions, closeness and distance and ICU nurses’ emotional responses, contributes to a discussion of embodied dignity as a coherent phenomenon through a framework with multiple variations of human dignity (Galvin & Todres, 2015).

Both patients and ICU nurses have emotional expressions. If the expressions in the eyes and face and the need for a change between closeness and distance are incorporated in building a mutual relationship, both partners’ dignity is maintained: “Dignity is the affirmation of something valuable in oneself or another as an ‘inheritor of Being’” (Galvin & Todres, 2015). In the interactive dialogue, eyes, facial reactions and other signals are interpreted as a need for a caring touch or to maintain distance. This interpretation is an affirmation and interpersonal recognition of the dignity of another human being. “Dignity thus lies in valuing this conjunction of the limits of being human and the immensity of being” (Galvin & Todres, 2015). Participant B in our study did get affected on sharing joys and sorrow with a stranger that connects people closely. This description of the conjunction of the patient’s vulnerability and value shows the preservation of mood and identity dignity in the nurses’ presence and experience and the actual meaning the ICU nurse gives to this experience. There are limitations; the nurse can bring interpersonal dignity to a patient’s situation when they share a presence and are on duty. However, the stability in the relationship requires a kind of primary care (Uhrenfeldt et al., 2018). When life itself is threatened, the ICU-nurse is honouring the patient by showing finitude dignity by giving a caring touch. By a caring touch, the ICU-nurse shows an embodied and relational understanding of the patient`s dignity.

Likewise, transfer of patients and time pressures in the ICU can be challenging both in understanding the patient expressions and responding in a caring and pleasant way (Martinsen, 2012). The ICU nurse may dwell on the patient’s and own expressions and accept both the emotional responses. It is a recognition of what it is to be a human being wherever you exist, as Galvin and Todres put it: “This essential conjunction of ‘vulnerability’ and ‘value’ participates in all modes of being-in-the-world whether it be an experience of self, or a perception of others or situations, or an activity that occurs in relation” (Galvin & Todres, 2015). This kind of dignity is easily ruptured or removed from the patient if a dehumanized attitude develops in ICU nurses due to their personality or working conditions, as argued by Participant B. They may be rushing along the bedsides without recognizing (maybe not wanting to) and addressing the patients’ needs, initiating treatment without warning or explanations or awaiting to see if the patients are ready. These situations are difficult for everyone; the patient may withdraw from ICU nurses, and the ICU nurse may feel ashamed of her (lack of) professional practice (Participant F).

In addition to procedures, technical skills and treatments, Scandinavian ICU nursing and caring touch are based on the patient’s wishes, as described by Orem in the 60s^22^ and further developed later by nursing theorists Benner & Wrubel, (2001); Martinsen, (1996) and Eriksson, (1995). Furthermore, the first six of seven kinds of dignity described above, encompasses the patient’s opportunity to reject the caring touch. Among many obligatory unpleasant examinations and treatments, the patient has the choice to reject the caring touch though it can provide a feeling of being respected and treated as a valuable human being in unpleasant surroundings (Galvin & Todres, 2015). ICU nurses describe signs of aggression (participant B, C, F), a “dark look” (participant C, F) and the patient pulling back in the bed (participant D) as signs to maintain distance. The ICU nurses show action that builds relationships when they try different touches and assess how the patient responds (participant G) and in this way carefully adjust their caring touch to each patient. In the worst case, it can feel as an abuse or assault if the nurse strokes the patient’s cheek if he does not want to, and there is a risk of losing dignity (Galvin & Todres, 2015)

Even though caring touch remains unexplored when it is without treatment aspect, it is considered an important nursing intervention as it is appraised even when the patient shows signs to maintain distance. The ICU nurses found that a gentle caring touch can help patients to express their feelings or talk about their worries (Participant A, E). Dignity seems to manifest as an embodied feeling (Galvin & Todres, 2015) in ICU nurses, a “gut feeling” (participant B), as they feel emotionally touched in the relationship with the patient.

In our study, patients expressed themselves in different ways, which invited ICU nurses to show dignity through caring touch. Simultaneously, ICU nurses showed awareness of the patient’s need for caring touch and the possibilities of preserving their dignity through it.

### Strengths and limitations

This study is significant in that it provides evidence for an understudied phenomenon. The strength lies in the dept of the conversation with the participants and the analysis, however, this phenomenon still needs further investigation. Both authors had an important participatory role in interpreting the data, which thus strengthens the trustworthiness of the study. The study follows a series of thorough academic critical steps and is therefore an expression of qualitative evidence.

It provides details based on experiences from the Scandinavian public healthcare system, with similarities to Benner and colleagues’ concept of clinical wisdom. To the best of our knowledge, this is the first paper focusing solely on caring touch and its significance for ICU patients. However, this study has some limitations. One limitation is the limited sample size and the single interview with each person. In a future study, a longitudinal range of interviews may bring additional valuable insights. However, the eight study participants provided a rich material that needed a purposeful selection to be presented in this paper. A limitation may be that the interviews were conducted in 2015 and then re-analysed in this article. However, the findings are found to be still relevant because there have been no or few changes when it comes to the Scandinavian health-care system and the ICU nurses` work tasks.

## Conclusion

Our new understanding is that the speaking body, which is our main result in this study, is presented as a motivating factor to give caring touch. Caring touch arises as a spontaneous action motivated by an overall sense that the patient`s dignity or hope may be threatened. Caring touch can, without specific purposes or actions, be an offer of individual dignity, an opening for hope in the midst of what may seem hopeless. Caring touch was early a well-known phenomenon in care theory, even though it has not been mentioned much in nursing theory for decades, until the latest ones. In this article, caring touch is a human way of being, that is grounded in the existential thinking and supported by an European theory of care where dignity is the central focal point.

From this study, we understood, that caring touch is a vital, but under-explored phenomenon in ICU nursing care. It is based on a compassionate, interpersonal and caring nurse-patient relationship and enhances the ethical dimension of dignity in intensive care nursing. Caring touch and patient’s dignity are phenomena that lack descriptions in intensive care units.

## References

[cit0001] Airosa, F., Arman, M., Sundberg, T., Öhlén, G., & Falkenberg, T. (2016). Caring touch as bodily anchor for patients after sustaining a motor accident with minor or no physical injuries - a mixed methods study. *BMC Complementary and Alternative Medicine*, 16(1), 106. 10.1186/s12906-016-1084-2 27004552 PMC4804542

[cit0002] Aitken, L. M., Marshall, A. P., Elliott, R., & McKinley, S. (2008). Critical care nurses’ decision making: Sedation assessment and management in intensive care. *Journal of Clinical Nursing*, 18(1), 36–12. 10.1111/j.1365-2702.2008.02318.x 18637856

[cit0003] Albarran, J., Rosser, E., Bach, S., Uhrenfeldt, L., Lundberg, P., & Law, K. (2011). Exploring the development of a cultural care framework for European caring science. *International Journal of Qualitative Studies on Health and Well-being*, 6(4), 10.3402/qhw.v6i4.11457. 10.3402/qhw.v6i4.11457 PMC3235943 22171224

[cit0004] Almerud, S., Alapack, R. J., Frilund, B., & Ekebergh, M. (2008). Beleaguered by technology, care in technologically intense environments. *Nursing Philosophy*, 9(1), 55–61. 10.1111/j.1466-769X.2007.00332.x 18154637

[cit0005] Arman, M., Ranheim, A., Rydenlund, K., Rytterström, P., & Rehnsfeldt, A. (2015). The Nordic tradition of caring science: The work of three theorists. *Nursing Science Quarterly*, 28(4), 288–296. 10.1177/0894318415599220 26396212

[cit0006] Benner, P., & Wrubel, J. (2001). *The primacy of caring: Stress and coping in health and illness*. Munksgaard.

[cit0007] Benner, P., Tanner, C. A., & Chesla, C. A. (2009). *Expertise in Nursing practice* (2nd ed.). Springer.

[cit0008] Benner, P., Hooper Kyriakides, P., & Stannard, D. (2011a). *Clinical wisdom and interventions in acute and critical care* (2nd ed.). Springer.

[cit0009] Benner, P., Hooper-Kyriakidis, P., & Stannard, D. (2011b). *Clinical wisdom and interventions in acute and critical care* (2nd ed.). Springer Publishing.

[cit0010] Borch, E., & Hillervik, C. (2005). Upplevelser av kroppslig beröring i omvårdnadsarbetet - patienter berättar (Experiences of physical touch in nursing care - narrated by patients). *Vård I Norden*, 25(4), 4–9. 10.1177/2F010740830502500402.

[cit0011] Bridges, J., Nicholson, C., Maben, J., Pope, C., Flatley, M., Wilkinson, C., Meyer, J., & Tziggili, M. (2013). Capacity for care: Meta‐ethnography of acute care nurses’ experiences of the nurse‐patient relationship. *Journal of Advanced Nursing*, 69(4), 760–772. 10.1111/jan.12050 23163719 PMC3617468

[cit0012] Bundgaard, K., & Nielsen, K. B. (2011). The art of holding hand. *International Journal of Human Caring*, 15(3), 34–41. 10.20467/1091-5710.15.3.34

[cit0013] Dahlberg, K., & Segesten, K. (2010). *Hälsa och vårdande: I teori och praxis*. Natur & kultur.

[cit0014] Dreyer, P. S., & Pedersen, B. D. (2009). Distanciation in Ricoeur’s theory of interpretation: Narrations in a study of life experiences of living with chronic illness and home mechanical ventilation. *Nursing Inquiry*, 16(1), 64–73. 10.1111/j.1440-1800.2009.00433.x 19228305

[cit0015] Eriksson, K. (1987). *Vårdandets idé (The concept of caring)*. Liber.

[cit0016] Eriksson, K. (1995). *Det lidende mennesket (The suffering human being)*. Tano AS.

[cit0017] European Academy of Caring Science (EACS). *Homepage*, Retrieved May 7, 2021, from www.eacs.nu

[cit0018] Fredriksen, S. T. D., & Ringsberg, K. C. (2007). Living the situation stress experiences among intensive care patients. *Intensive and Critical Care Nursing*, 23(3), 124–131. 10.1016/j.iccn.2006.09.002 17088063

[cit0019] Galvin, K. T. (2010). Revisiting caring science: Some integrative ideas for the ‘head, hand and heart’ of critical care nursing practice. *Nursing in Critical Care*, 15(4), 168–175. 10.1111/j.1478-5153.2010.00394.x 20626793

[cit0020] Galvin, K., & Todres, L. (2015). Dignity as honour-wound: An experiential and relational view. *Journal of Evaluation in Clinical Practice*, 21(3), 410–418. 10.1111/jep.12278 25345355 PMC4674983

[cit0021] Ghezeljeh, T. N., Farahani, M. A., & Ladani, F. K. (2021). Factors affecting error communication in intensive care units: A qualitative study. *Nursing Ethics*, 28(1), 131–144. 10.1177/0969733020952100 32985367

[cit0022] Gleeson, M., & Timmins, F. (2005). A review of the use and clinical effectiveness of touch as a nursing intervention. *Clinical Effectiveness in Nursing*, 9(1–2), 69–77. 10.1016/j.cein.2004.12.002

[cit0023] Heaton, J. (2008). Secondary analysis of qualitative data: An overview. *Historical Social Science*, 33(3), 33–45. https://www.jstor.org/stable/20762299.

[cit0024] Henderson, V. (1991). *The nature of nursing. Reflections after 25 years*. National League for Nursing. 1780233

[cit0025] Henderson, V. (1997). *Sykepleiens grunnprinsipper (Basic principles of nursing care)*. Norsk Sykepleierforbund.

[cit0026] Hov, R., Hedelin, B., & Athlin, E. (2007). Good nursing care to ICU patients on the edge of life. *Intensive and Critical Care Nursing*, 23(6), 331–341. 10.1016/j.iccn.2007.03.006 17689250

[cit0027] Kvale, S., & Brinkmann, S. (2009). *Det kvalitative forskningsintervju (The qualitative interview)*. Gyldendal akademisk.

[cit0028] Lindseth, A., & Norberg, A. (2004). A phenomenological hermeneutical method for researching lived experience. *Scandinavian Journal of Caring Sciences*, 18(2), 145–153. 10.1111/j.1471-6712.2004.00258.x 15147477

[cit0029] Lindström, U. Å., Nyström, L. L., & Zetterlund, J. E. *Theory of caritative caring.* In Nursing theorists and their work. Alligood, M.R. (red.) 9th ed. (2019). Chpt 11. Retrieved May 7, 2021, Chpt 11. https://nursekey.com/11-theory-of-caritative-caring/

[cit0030] Magro-Morillo, A., Boulayoune-Zaagougui, S., Cantón-Habas, V., Molina-Luque, R., Hernández-Ascanio, J., & Ventura-Puertos, P. E. (2020). Emotional universe of intensive care unit nurses from Spain and the UK: A hermeneutic approach. *Intensive and Critical Care Nursing*, *9*(58), 102850. 10.1016/j.iccn.2020.102850 32229184

[cit0031] Martinsen, K., (Ed.) (1993). *Den omtenksomme sykepleier*. TANO.

[cit0032] Martinsen, K. (1996). *Fenomenologi og omsorg (Phenomenology and care)*. Tano Aschehoug.

[cit0033] Martinsen, K. (2012). *Løgstrup og sykepleien (Løgstrup and nursing)*. Akribe AS.

[cit0034] Maslow, A. (1943). A theory of human motivation. *Psychological Review*, 50(4), 370–396. 10.1037/h0054346

[cit0035] Moitra, V. K., Guerra, C., Linde-Zwirble, W. T., & Wunsch, H. (2016). Relationship between ICU length of stay and long-term mortality for elderly ICU survivors. *Critical Care Medicine*, 44(4), 655–662. 10.1097/CCM.0000000000001480 26571190 PMC4792682

[cit0036] Nist, M. D., Harrison, T. M., Tate, J., Robinson, A., Balas, M., & Pickler, R. H. (2020). Losing touch. *Nursing Inquiry*, 27(3), 1–3. 10.1111/nin.12368 32697024

[cit0037] Orem, D. (2001). *Nursing: Concepts of practice* (6th ed.). Mosby.

[cit0038] Ozolins, -L.-L., Hörberg, U., & Dahlberg, K. (2015). Caring touch: Patients' experiences in an anthroposophic clinical context. *Scandinavian Journal of Caring Sciences*, 29(4), 834–842. 10.1111/scs.12242 26178972

[cit0039] Parola, V. Coalho, A., Fernandes, O., & Apostle, J. (2020). Travelbee’s Theory: Human-to-human relationship model - its suitability for palliative nursing care. *Revista de Enfermagem Referência*, V(2), 1–7. http://hdl.handle.net/10400.26/37581.

[cit0040] Playfare, C. (2010). Human relationships: An exploration of loneliness and touch. *The British Journal of Nursing*, 19(2), 124–126. doi:10.12968/bjon.2010.19.2.46301. 20220652

[cit0041] Ricoeur, P. (1999). *Eksistens og hermeneutikk (Explanation and understanding)*. Skien, Aschehoug & co.

[cit0042] Tønnessen, S., Scott, A., & Nortvedt, P. (2020). Safe and competent nursing care: An argument for a minimum standard? *Nursing Ethics*, 27(6), 1396–1407. 10.1177/0969733020919137 32419621 PMC7543010

[cit0043] Travelbee, J. (1971). *Interpersonal aspects of nursing*. FA Davis Company.

[cit0044] Uhrenfeldt, L. *Sygepleje i samspil med fænomenologi og videnskab. (Nursing in interaction with phenomenology and science)Master thesis Aarhus University*, 1996, Retrieved May 7, 2021, from https://www.researchgate.net/publication/316967566_Sygepleje_i_samspil_med_faenomenologi_og_videnskab_En_undersogelse_af_det_grundlag_Watson_Martinsen_og_Eriksson_har_for_kaerlig_omsorg_i_sygeplejen

[cit0045] Uhrenfeldt, L., Sørensen, E. E., Bahnsen, I. B., & Pedersen, P. U. (2018). The centrality of the nurse–patient relationship: A Scandinavian perspective. *Journal of Clinical Nursing*, 27(15–16), 3197–3204. 10.1111/jocn.14381 29633404

[cit0046] Usberg, G., Uibu, E., Urban, R., & Kangasniemi, M. (2021). Ethical conflicts in nursing: An interview study. *Nursing Ethics*, 28(2), 230–241. 10.1177/0969733020945751 32909885

[cit0047] World Medical Association. 2013. *The Helsinki declaration*, Retrieved June 29, 2021, from www.wma.net

